# Biocontrol of tomato bacterial wilt by the new strain *Bacillus velezensis* FJAT-46737 and its lipopeptides

**DOI:** 10.1186/s12866-020-01851-2

**Published:** 2020-06-15

**Authors:** Meichun Chen, Jieping Wang, Bo Liu, Yujing Zhu, Rongfeng Xiao, Wenjing Yang, Cibin Ge, Zheng Chen

**Affiliations:** 1 Agricultural Bioresources Research Institute, Fujian Academy of Agricultural Sciences, Fuzhou, 350003 China; 2 College of Biological Science and Engineering, Fuzhou University, Fuzhou, 350001 China

**Keywords:** *Bacillus velezensis* FJAT-46737, Lipopeptides, Antagonistic activities, Bacterial wilt, Fengycin, Culture conditions

## Abstract

**Background:**

There is an urgent need to discover biocontrol agents to control bacterial wilt. This study reports on a new lipopeptide-producing biocontrol strain FJAT-46737 and explores its lipopeptidic compounds, and this study investigates the antagonistic effects of these compounds.

**Results:**

Based on a whole genome sequence analysis, the new strain FJAT-46737 was identified as *Bacillus velezensis*, and seven gene clusters responsible for the synthesis of bioactive secondary metabolites in FJAT-46737 were predicted. The antimicrobial results demonstrated that FJAT-46737 exhibited broad-spectrum antimicrobial activities in vitro against three bacteria and three fungi. Pot experiments showed that the control efficiencies for tomato bacterial wilt of the whole cultures, the 2-fold diluted supernatants and the crude lipopeptide of FJAT-46737 were 66.2%, 82.0%, and 96.2%, respectively. The above results suggested that one of the antagonistic mechanisms of FJAT-46737 was the secretion of lipopeptides consisting of iturins, fengycins and surfactins. The crude lipopeptides had significant antagonistic activities against several pathogens (including *Ralstonia solanacearum*, *Escherichia coli* and *Fusarium oxysporum*) and fengycins were the major antibacterial components of the lipopeptides against *R. solanacearum* in vitro. Furthermore, the rich organic nitrogen sources (especially yeast extracts) in the media promoted the production of fengycin and surfactin by FJAT-46737. The secretion of these two lipopeptides was related to temperature fluctuations, with the fengycin content decreasing by 96.6% and the surfactins content increasing by 59.9% from 20 °C to 40 °C. The optimal temperature for lipopeptide production by FJAT-46737 varied between 20 °C and 25 °C.

**Conclusions:**

The *B. velezensis* strain FJAT-46737 and its secreted lipopeptides could be used as new sources of potential biocontrol agents against several plant pathogens, and especially the bacterial wilt pathogen *R. solanacearum*.

## Background

Bacterial wilt caused by *Ralstonia solanacearum* is a devastating disease that affects almost 250 plant species, and it seriously threaten plant growth and leads to huge losses worldwide [1, 2]. Traditional agricultural practices, such as crop rotation, field sanitation, resistant variety cultivation, and chemical bactericide application, have been widely applied to control the bacterial wilt, although they present certain shortcomings [3]. The long-term or excessive use of chemical bactericides caused pathogen resistance and has adverse effects on the environment, beneficial organisms, and human health [3]. The identification of safe, effective and alternative methods for controlling plant pathogen diseases is becoming increasingly important for improving the output and quality of agricultural products. Furthermore, biological control using antagonistic microorganisms including bacteria, yeasts, and filamentous fungi, represent a safe, effective and sustainable alternative method for fighting plant pathogens compared with chemical bactericides [4–6]. Members of the genus *Bacillus* have been used as effective biocontrol agents to reduce damages caused by bacterial wilt, and their derived products account for approximately half of the commercially available biopesticides [7]. However, the control of bacterial wilt is difficult and ineffective due to the high genetic variability, persistence in the environment and broad host range of *R. solanacearum*. Thus, the use of alternative antimicobial agents is needed [3, 8].

Studies on the biocontrol mechanisms of *Bacillus* agents indicated that the biocontrol effects of certain strains are primarily associated with their production of various bioactive molecules [9]. Lipopeptides belong to the most important bioactive substances and they exhibit excellent properties, such as broad-spectrum antibiotic activity, good stability, low toxicity, high biodegradability, and reduced drug resistance susceptibility [10]. *Bacillus* lipopeptides include three classes, namely, iturin (bacillomycin D/F/L/Lc, iturin A/C/D/E, and mycosubtilin), fengycin (fengycin A/B, and plipastatin A/B) and surfactin (halobacillin, pumilacidin and surfactin) [11–13]. All the mentioned classes share a common amphiphilic molecule structure composed of a fatty acid side chain linking to a cyclic peptide ring (Figure S1). Among them, the iturins exhibit strong antifungal activities against various types of yeast and filamentous fungi but have limited antibacterial activity; the fengycins have strong antifungal activities, especially on filamentous fungi, although their ability to inhibit bacteria has only recently been reported by Villegas-Escobar et al. [14]; and the surfactins display effective bactericidal activities and can reduce the surface tension of plant roots, which facilitates the swimming ability and biofilm formation of *Bacillus* strains, thereby providing protection against pathogen attacks [15].

Studies of lipopeptides have mainly focused on the control of plant fungal pathogens, such as *Rhizoctonia solani* [16], *Pythium ultimum* [4], *Botrytis cinerea* [17], *Podosphaera fusca* [17], *Fusarium graminearum* [18, 19], *Fusarium oxysporum* [19], and *Sclerotinia sclerotiorum* [20]. Few studies have focused on *R. solanacearum* bacterial pathogens. Zhu et al. [21] reported that the lipopeptide mixture (surfactin and iturin A) produced by *Bacillus amyloliquefaciens* XZ-173 could effectively inhibit the growth of *R. solanacearum*. Furthermore, the lipopeptides secreted by XZ-173 were used to manufacture lipopeptide-mineral composites to achieve 87.76% biocontrol efficacy on bacterial wilt of tomato [22]. Lipopeptides suppress plant diseases either by impeding the pathogens directly or by promoting induced systemic resistance in the host plants [17, 23, 24]. A strong correlation has been reported between defense-inducing activity and the surfactin content generated by *Bacillus* strains [25]. In addition, it has been reported that lipopeptides secreted by *Bacillus* bacteria depend on the strain it self, the culture medium components and cultivation conditions [26–28].

The present study reports on the new lipopeptide-producing biocontrol strain FJAT-46737, which was identified via a whole genome sequence analysis, and the bioactive secondary metabolite gene clusters in the strain were predicted. The suppressive effects of FJAT-46737 and its lipopeptides were evaluated in vitro and in vivo. Furthermore, the lipopeptide components were identified; and effects of the culture conditions on the antibacterial activity of the strain and its lipopeptides were investigated. This work tend to promote the application of strain FJAT-46737 and its lipopeptides as new sources of biocontrol agents against plant pathogens, especially the bacterial wilt pathogen *R. solanacearum*.

## Results

### Identification and antagonistic activities of FJAT-46737

Strain FJAT-46737 is a gram-positive and endospore-forming bacterium. Its colonies on the NA plate were flat, slightly rough, nearly circular, and light yellow (Figure S2). The phylogenetic analysis of the 16*S rRNA* and *gyrB* gene results indicated that strain FJAT-46737 belongs to the genus *Bacillus* and is closely related to strains *B. velezensis* and *B. amyloliquefaciens* (Figure S3, S4). The *16 S rRNA* gene of FJAT-46737 exhibits 99.72, 99.71 and 99.44% similarity to the type strains *Bacillus siamensis* KCTC 13613^T^, *Bacillus velezensis* CR-502^T^ and *B. amyloliquefaciens* DSM7^T^, respectively. The *gyr B* gene of FJAT-46737 displayed greater than 99.0% identity with the type species of *B. amyloliquefaciens* and *B. velezensis*. Because of the high degree of relatedness among *B. amyloliquefaciens*, *B. siamensis* and *B. velezensis*, the ANI method based on the whole-genome sequence was used to discriminate strain FJAT-46737.

The whole-genome sequence of strain FJAT-46737 contains 3,995,340 bp, and the G + C content of the chromosomal DNA was 46.5%. The ANI values between the strain FJAT-46737 and the type strains *B. amyloliquefaciens* DSM7^T^, *B. siamensis* KCTC 13613^T^, and *B. velezensis* KCTC 13012^T^ were calculated as 94.16, 94.36 and 98.26%, respectively. The last one displayed ANI values of > 98%, which exceeded the recommended cut-off of 96% for species delineation. This result suggested that strain FJAT-46737 was a member of *B. velezensis* species.

The gene clusters of bioactive secondary metabolites in strain FJAT-46737 were analyzed using the antiSMASH method. The results have shown that strain FJAT-46737 possesses 7 gene clusters that are responsible for the synthesis of surfactin, fengycin, macrolactin, bacillaene, difficidin, bacilysin and bacillibactin (Figure S5). These findings indicate that strain FJAT-46737 presents strong antagonistic activities.

**Fig. 1:**
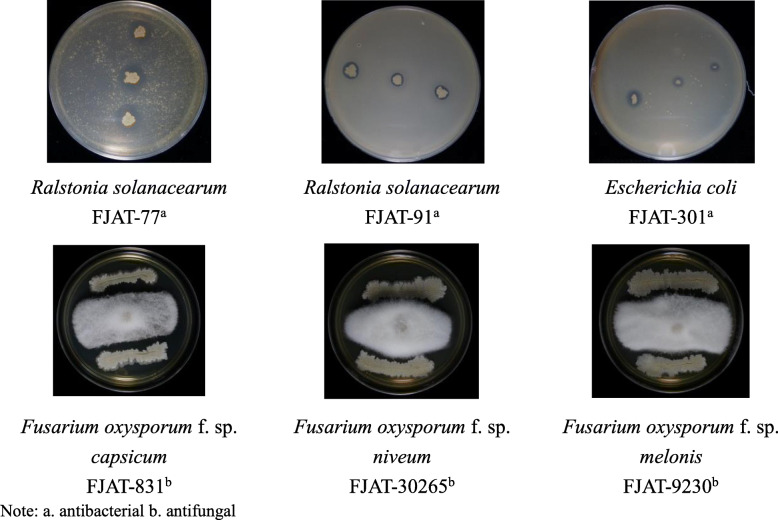
The spectrum of antimicrobial activity of FJAT-46737 against bacteria and filamentous fungi

The agar disk diffusion method was used to evaluate the antagonistic activities of FJAT-46737 against several animal or plant pathogens. The results showed that this strain exhibited significant activities against gram-negative bacteria, including *R. solanacearum* and *E. coli* with the inhibition zone diamiter of 6.95–14.56 mm, and filamentous fungi, including 3 biotypes of. *oxysporum* (*F. oxysporum* f. sp. *capsicum*, *F. oxysporum* f. sp. *niveum* and *F. oxysporum* f. sp. *melonis*) (Fig. 1) with the inhibition rate of 65.25%–72.71%.

### Biocontrol efficacy of FJAT-46737 against tomato bacterial wilt

**Fig. 2:**
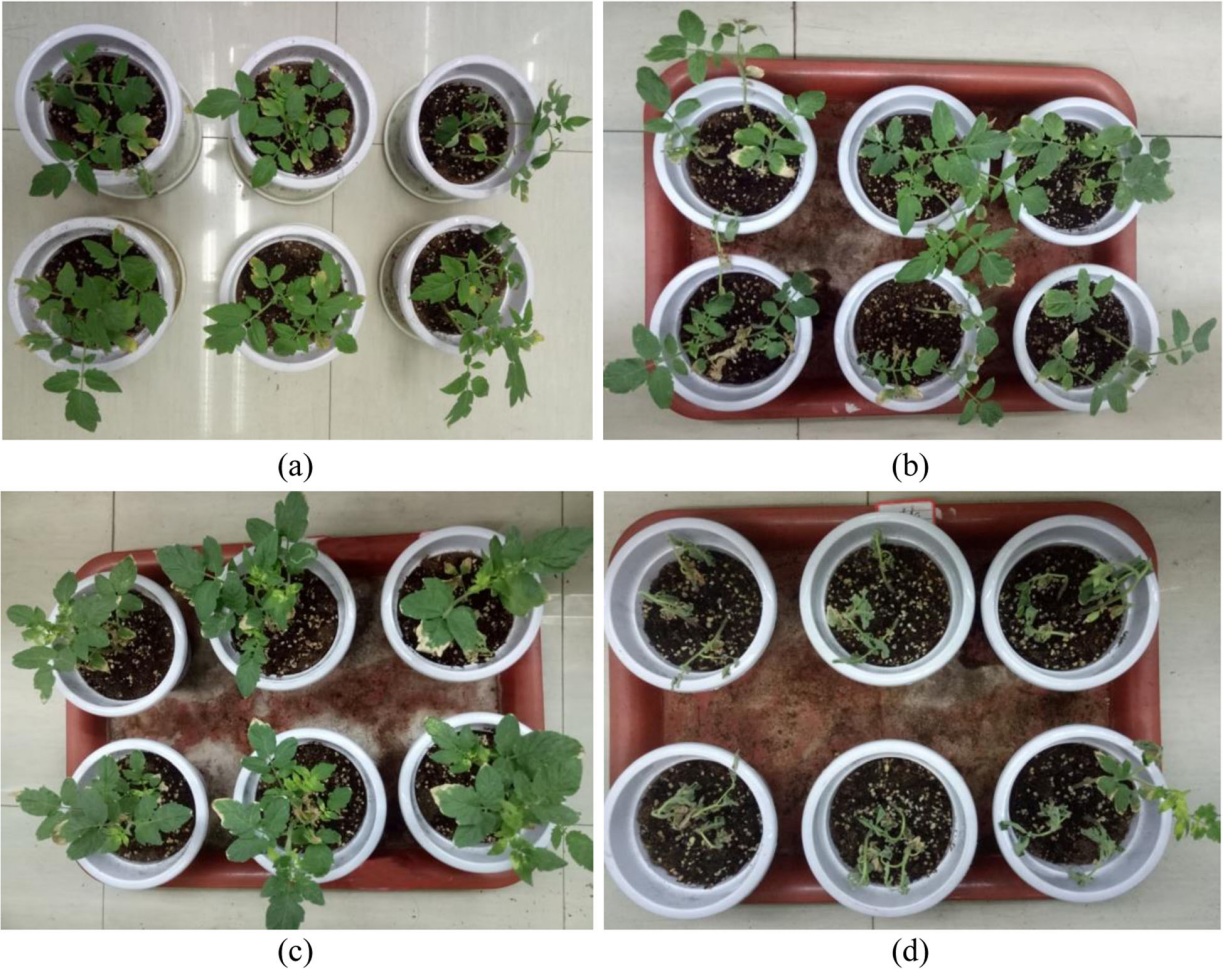
Biocontrol effect of the whole cultures of FJAT-46737 (**a**), the culture supernatant (**b**), 1 mg/mL crude lipopeptides (**c**), and control (**d**) against bacterial wilt disease. Note: a. antibacterial b. antifungal

The above results showed that the *B. velezensis* strain FJAT-46737 had in vitro antibacterial activity against *R. solanacearum*. Thus, we attempted to investigate its biocontrol efficacy against tomato bacterial wilt by performing pot experiments under greenhouse conditions. First, we found that the DI of the tomato plants in the treatment group with whole cultures of FJAT-46737 (31.7%) was much lower than that of the control group (93.8%) (Fig. 2, Table 1). The biocontrol efficacy of whole cultures of FJAT-46737 against tomato bacterial wilt could reach 66.2% in the greenhouse experiments (Table 1).

**Table 1:** The biocontrol efficacies and the disease incidence in differently treatment groups of plants

Treatment group	biocontrol efficiency	Disease incidence (DI)
Control group	0%	93.8%
Whole culture	66.2%	31.7%
2-fold diluted cell-free supernatants	82.0%	17.5%
Lipopeptide (1 mg/mL)	96.2%	3.7%

To clarify the biocontrol mechanisms of FJAT-46737, we subsequently evaluated the suppressive effects of its cell-free supernatants on tomato bacterial wilt. A preliminary experiment indicated that undiluted cell-free supernatant caused seedling injury while the 2-fold diluted supernatant had no injury effect. Thus, the 2-fold diluted cell-free supernatants was selected to perform the pot experiments. The results showed that an approximately 82.0% biocontrol efficiency could be achieved in the cell-free supernatant treatment group (Fig. 2, Table 1), which indicated that the biocontrol ability of FJAT-46737 could be mainly (if not entirely) attributed to its production of extracellular bioactive substances.

We further tested the biocontrol efficiency of the crude lipopeptide extracts from FJAT-46737 against tomato root infection by *R. solanacearum*. A preliminary experiment indicated that a high concentration (≥2.5 mg/mL) of lipopeptides led to seedling injury while 1 mg/ml lipopeptides had no injury effect. Thus, a concentration of 1 mg/mL lipopeptides was selected to perform the pot experiments. The treatment in which plantlets were soaked with the lipopeptides could significantly reduce the mortality of the tomato plants and achieve a biocontrol efficiency of 96.2% (Fig. 2, Table 1). These results suggested that one of the antagonistic mechanisms of strain FJAT-46737 is the secretion of lipopeptides.

### LC-QTOF-MS/MS analyses of the lipopeptides produced by FJAT-46737

In this study, we used the LC-ESI-MS/MS method to determine the lipopeptide profile of FJAT-46737. Three classes of cyclic lipopeptides, namely, iturin (retention time, 12.8–22.3 min), fengycin (29.0–36.0 min), and surfactin (48.3–52.0 min), were detected, and the retention times, MS and MS^2^ spectral data and identification results for the lipopeptides from *B. velezensis* FJAT-46737 are summarized in Table 2. The results show that the lipopeptides consisted of C_14_–C_16_ iturin A, C_13_–C_15_ surfactins, and C_16_ fengycin A/B and C_16_ fengycin A_2_/B_2_ .

**Table 2:** Identification of lipopeptides extracted from strain *Bacillus velezensis* FJAT-46737 through LC-QTOF-MS/MS

Retention time (min)	MS m/z [M + H]^+^ /[M + Na]^+^	Identified Compounds
12.833	1065.5^a^	C_14_Iturin A
16.632	1079.7^a^	C_15_Iturin A
17.536	1079.7^a^	C_15_Iturin A
21.244	1093.6^a^	C_16_Iturin A
22.284	1093.6^a^	C_16_Iturin A
29.067	1449.9^b^	C_16_FengycinA_2_
30.423	1449.9^b^	C_16_FengycinA_2_
30.514	1463.9^b^	C_16_FengycinA
31.056	1463.9^b^	C_16_FengycinA
31.735	1449.9^b^	C_16_FengycinA_2_
32.413	1477.9^b^	C_16_FengycinB_2_
33.182	1463.9^b^	C_16_FengycinA
34.132	1477.9^b^	C_16_FengycinB_2_
34.991	1477.9^b^	C_16_FengycinB_2_
35.262	1491.8^b^	C_16_FengycinB
35.986	1491.8^b^	C_16_FengycinB
48.376	1030.8^a^	C_13_Surfactin
50.26	1044.8^a^	C_14_Surfactin
50.757	1044.8^a^	C_14_Surfactin
51.345	1030.8^a^	C_13_Surfactin
51.978	1058.8^a^	C_15_Surfactin

Note: ^a^ is [M + Na]^+^ and ^b^ is [M + H]^+^

### Inhibitory spectra of the antagonistic lipopeptides

We used an agar well diffusion assay to further evaluate the antimicrobial activities of the crude lipopeptides from FJAT-46737. The results showed that the crude lipopeptides had significant activities against *R. solanacearum*, *E. coli*, and *F. oxysporum* in a dosage-dependent manner (Tables 3 and 4). At 48 h, the inhibition zone diameters under treatment with 10 mg/mL of the crude lipopeptides could reach up to 18.52 ± 0.73 mm, 14.55 ± 0.23 mm, and 14.57 ± 1.85 mm against the pathogens *R. solanacearum* FJAT-91 and FJAT-77, and *E. coli* FJAT-301, respectively (Table 3). The lipopeptide concentration of 0.5 mg/mL displayed antibacterial activity against *R. solanacearum* FJAT-91, while the concentration 0.25 mg/mL did not (Figure S6). These results implied that the crude lipopeptides of FJAT-46737 had the strongest antibacterial activity on *R. solanacearum*. Moreover, under 30 mg/mL of the crude lipopeptides, the inhibition zone diameters for 4 biotypes of *F. oxysporum* (*F. oxysporum* f. sp. *capsicum* FJAT-831, *F. oxysporum* f. sp. *niveum* FJAT-9230 and *F. oxysporum* f. sp. *melonis* FJAT-30265) were all approximately 20 mm at 72 h.

**Table 3:** Antibacterial ability of lipopeptides from strain *Bacillus velezensis* FJAT-46747

		Diameter of inhibition zone (mm)		
indicator strains		*Ralstonia solanacearum* FJAT-91 (tomato pathogens)	*Escherichia coli* FJAT-301	*Ralstonia solanacearum* FJAT-77 (peanut pathogens)
concentration of crude lipopeptides (mg/mL)	10	18.52 ± 0.73^*a*^	14.55 ± 0.23^*a*^	14.57 ± 1.85^*a*^
	5	16.04 ± 0.26^*b*^	12.32 ± 1.14^*b*^	13.90 ± 0.43^*a*^
	2.5	13.87 ± 0.99^*b*^	10.02 ± 0.01^*c*^	11.19 ± 0.03^*a*^
	1	12.51 ± 0.58^*b*^	weak	–

Note: Values were expressed as mean ± standard deviation (*n* = 3)

The difference letter in the same column indicated that the difference between the grades is significantly through Duncan test (*p* < 0.05)

**Table 4:** Antifungal ability of lipopeptides from strain *Bacillus velezensis* FJAT-46747

		Diameter of inhibition zone (mm)		
indicator strains		*Fusarium oxysporum* f. sp. *capsicum* FJAT-831	*Fusarium oxysporum* f. sp. *melonis* FJAT-9230	*Fusarium oxysporum* f. sp. *niveum* FJAT-30265
concentration of crude lipopeptides (mg/mL)	30	17.87 ± 0.11^*a*^	18.77 ± 0.25^*a*^	20.47 ± 3.08^*a*^
	20	16.62 ± 0.78^*a*^	18.36 ± 0.44^*a*^	17.28 ± 0.64^*a*^
	10	13.84 ± 0.58^*b*^	16.00 ± 0.22^*b*^	14.51 ± 1.35^*a*^

Note: Values were expressed as mean ± standard deviation (*n* = 3)

The difference letter in the same column indicated that the difference between the grades is significantly through Duncan test (*p* < 0.05)

### Effects of medium components and culture conditions on the antibacterial activities and contents of lipopeptides

To further improve the antagonistic activities of FJAT-46737, we analyzed the effects of the medium components and temperatures on the antibacterial activities of the cluture supernatants and crude lipopeptides. Given that the crude lipopeptides of FJAT-46737 had strong antibacterial activity on *R. solanacearum*, FJAT-91 was used as the indicator bacterium. The results of the antagonistic activity of the supernatant, crude lipopeptides and lipopeptide contents under different conditions are shown in Tables 5 and 6.

**Table 5:** Diameter of inhibition zone of the culture supernatant and crude lipopeptides (10 mg/mL) produced by FJAT-46737 against *Ralstonia solanacearum* FJAT-91 and the content of lipopeptide under different culture medium

	Medium	A	B	C	D	E	F
Diameter of inhibition zone (mm)	supernatant	–	–	15.75 ± 0.38^*ab*^	14.93 ± 0.77^*b*^	16.36 ± 0.60^*a*^	12.77 ± 0.88^*c*^
	Lipopeptides	13.88 ± 2.54^*b*^	–	21.41 ± 1.60^*a*^	19.15 ± 0.78^*a*^	19.75 ± 0.93^*a*^	15.67 ± 1.00^*b*^
Yield of lipopeptide in the supernatant (mg/L)	iturin	0.53 ± 0.20^*cd*^	0.31 ± 0.09^*d*^	0.98 ± 0.02^*b*^	0.68 ± 0.14^*c*^	1.99 ± 0.18^*a*^	0.38 ± 0.02^*d*^
	fengycin	22.17 ± 11.94^*b*^	9.03 ± 1.69^*b*^	58.66 ± 9.81^*a*^	50.37 ± 12.46^*a*^	24.34 ± 2.75^*b*^	9.17 ± 1.21^*b*^
	surfactin	0.65 ± 0.42^*a*^	0.55 ± 0.23^*a*^	1.39 ± 0.06^*a*^	1.21 ± 0.35^*a*^	0.61 ± 0.06^*a*^	0.59 ± 0.13^*a*^
	Total lipopeptides	23.34 ± 12.54^*b*^	10.07 ± 1.80^*b*^	61.04 ± 9.86^*a*^	52.27 ± 12.70^*a*^	27.54 ± 3.90^*b*^	10.22 ± 1.29^*b*^

Note: Values were expressed as mean ± standard deviation (*n* = 3)

The difference letter in the same row indicated that the difference between the grades is significantly through Duncan test (*p* < 0.05). The effects of medium components on the antibacterial activities and contents of lipopeptides were carried out at 25 °C

**Table 6:** Diameter of inhibition zone of the culture supernatant and crude lipopeptides (10 mg/mL) produced by FJAT-46737 against *Ralstonia solanacearum* FJAT-91 and the content of lipopeptide under different culture temperature

	Temperature	20 °C	25 °C	30 °C	35 °C	40 °C
Diameter of inhibition zone (mm)	supernatant	12.36 ± 0.46^*b*^	14.47 ± 1.06^*a*^	12.06 ± 0.26^*b*^	–	–
	Lipopeptides	14.34 ± 0.90^*b*^	17.16 ± 0.46^*a*^	15.53 ± 1.83^*ab*^	11.00 ± 0.49^*c*^	–
Yield of lipopeptide in the supernatant (mg/L)	iturin	0.65 ± 0.03^*b*^	0.78 ± 0.07^*a*^	0.86 ± 0.10^*a*^	0.61 ± 0.07^*b*^	0.45 ± 0.01^*c*^
	fengycin	55.11 ± 4.74^*a*^	56.47 ± 6.59^*a*^	23.62 ± 6.34^*b*^	9.55 ± 0.27^*c*^	1.88 ± 0.28^*c*^
	surfactin	0.59 ± 0.06^*b*^	0.83 ± 0.19^*b*^	0.78 ± 0.13^*b*^	1.22 ± 0.21^*a*^	1.47 ± 0.15^*a*^
	Total lipopeptides	56.30 ± 4.76^*a*^	57.94 ± 4.76^*a*^	25.41 ± 6.37^*b*^	11.39 ± 0.53^*c*^	3.72 ± 0.36^*c*^

Note: Values were expressed as mean ± standard deviation (*n* = 3)

The difference letter in the same row indicated that the difference between the grades is significantly through Duncan test (*p* < 0.05). The effects of temperature on the antibacterial activities and contents of lipopeptides were carried out using the culture media C

Using the selected culture media A ~ F (Table S1), the supernatants and crude lipopeptides of FJAT-46737 exhibited good antibacterial activities against *R. solanacearum* FJAT-91, except for the culture supernatant from the medium A (Table 5). Moreover, the antibacterial activities of both the supernatants and crude lipopeptides from culture media C, D and E were stronger than those from culture media A, B and F (Table 5). These results suggested that the medium components could significantly affect the antibacterial activities of FJAT-46737.

Subsequently, the effects of temperature on the antibacterial activities of the cell-free supernatants and crude lipopeptides were determined using the culture medium C. The results showed that the culture temperature had significant effects on the antibacterial activities of the cell-free supernatants and crude lipopeptides: i) the cell-free supernatants did not exhibit antibacterial activity when the temperature was higher than 35 °C; and ii) both the cell-free supernatants and crude lipopeptides had the strongest antibacterial activities when incubated at 25 °C (Table 6).

The iturin, fengycin and surfactin contents in different culture conditions were further determined by LC-QTOF-MS and are summarized in Tables 5 and 6. The fengycins were the most abundant lipopeptide family produced by FJAT-46737. The iturin, fengycin and surfactin contents in the six culture media varied within the range of 0.31–1.99 mg/L, 9.03–58.66 mg/L and 0.55–1.39 mg/L, respectively. The contents of lipopeptides in culture media C and D were significantly higher than those in the other four media, indicating that media containing a rich organic nitrogen source might be benificial for lipopeptides production. Moreover, adding yeast extract in culture medium D significantly increased the yield of lipopeptides compared with the lack of yeast extract (in culture medium B). In culture medium C, the production of lipopeptides (61.04 ± 9.86 mg/L) was the highest compared to the other media. Moreover, the contents of the iturins, fengycins and surfactins were strongly dependent on temperature. The contents of fengycins decreased by 96.6% when the culture temperature increased from 20 °C to 40 °C while contents of surfactins increased by 59.9%. The temperature ranges of 25–30 °C, 20–25 °C and 35–40 °C were suitable for the production of iturin, fengycin and surfactin, respectively.

Subsequently, a correlation analysis was performed between the cell-free supernatant antibacterial activities and lipopeptide contents in different culture conditions (Table 7). The contents of fengycins and total lipopeptides were significantly correlated with the antibacterial activities of the cell-free supernatants of all samples (*p* < 0.05), whereas the contents of surfactins and iturins did not show this correlation. These results suggested that the antibacterial activities of the cell-free supernatants were mainly attributed to the secretion of the fengycins by FJAT-46737.

**Table 7:** Correlation coefficients between antibacterial activities of the cultivation supernatant and the lipopeptide contents

	Pearson correlation coefficient	Kendall correlation coefficient	Spearman correlation coefficient
Iturin	0.570	0.597^*^	0.740^*^
Fengycin	0.695^*^	0.559^*^	0.726^*^
Surfactin	−0.100	0.078	0.033
Total lipopeptide	0.705^*^	0.559^*^	0.726^*^

Test of significance by two-tailed

*p < 0.05

### Effect of purified lipopeptides on the inhibition of *R. solanacearum* growth

The antibacterial activities of the purified lipopeptides against *R. solanacearum* were tested. The results showed that only the fraction obtained by SPE with 70% MeOH (named SPE70) exhibited antibacterial activity. Therefore, the composition of the fraction SPE70 was further determined by LC-QTOF-MS/MS (Figure S7). The results showed that the fraction SPE70 contained only fengycin, indicating that fengycin plays an important role in the growth inhibition of *R. solanacearum*. This result was consistent with the above correlation analysis.

## Discussion

Traditional phenotypic methods and phylogenetic analysis of conserved gene are insufficient to distinguish the species of *Bacillus*, including *B. subtilis*, *B. amyloliquefaciens*, *B. velezensis*, *B. siamensis*, *B. licheniformis*, and *B. pumilus*, due to the close correlations between these species [29]. However, the increased availability of complete genome sequences has facilitated the discrimination of these species. The ANI value based on the complete genome sequences was calculated, and a cut-off of 96% was proposed for species delineation [29, 30]. In the present study, strain FJAT-46737 displayed 98.26% similarity to the *B. velezensis* type strain KCTC 13012^T^, which is well above of the recommended threshold of 96% for species delineation. Thus, FJAT-46737 was identified as *B. velezensis*.

The occurrence of bacterial wilt in crops has led to great economic losses wordwide. Chemicals are often used in controlling plant diseases, although they lead to environmental pollution and microbial pathogen resistance [3]. Many studies have shown that the use of *Bacillus* strains as biological control agents is as a promising and safe strategy for effective tomato bacterial wilt management. Kwon and Kim reported that *B. subtilis* JW-1 could lead to a > 80% reduction in bacterial wilt disease and can be used as a potential biocontrol agent of tomato bacterial wilt [31]. Xiong et al. isolated the *B*. *amyloliquefaciens* JK6 strain and found that it could effectively suppress tomato bacterial wilt with biocontrol efficacies for up to 52.9% under two greenhouse conditions [32]. *B. subtilis* 916 exhibited biocontrol efficiency of 55.6% on tomato bacterial wilt [33]. In the present study, we identified a new biocontrol strain *B. velezensis* FJAT-46737, and it exhibits broad-spectrum antimicrobial activities against gram-negative bacteria and filamentous fungi. The biocontrol efficacy of strain FJAT-46737 against tomato bacterial wilt was 66.2%, which was higher than that of many previously reported *Bacillus* spp. strains, such as the strains APF1 (60.3%) [34], JK6 (58.6%) [32] and 916 (55.6%) [33].

The biocontrol ability of *Bacillus* strains against plant pathogens has been confirmed to be achieved by the induction of host systemic resistance, antibiotic production, siderophore and lytic enzymes secretion, biofilm formation, or niches competition within the rhizosphere [35]. Bais et al. reported that *B. subtilis* 6051 was able to control *Pseudomonas syringae* root infection in *Arabidopsis* because of the secretion of surfactin and the formation of a biofilm on the plant roots [15]. Kwon and Kim demonstrated that *B. subtilis* JW-1 induced a significant bacterial wilt disease suppression effect in vivo due to the production of cyclic lipopeptides [31]. Xiong et al. reported that the secretion of surfactin by the strain JK6 played important roles in the biocontrol of tomato bacterial wilt [32]. In the present study, the biocontrol efficiency of the cell-free supernatant against tomato bacterial wilt was 82.0% while that of the lipopeptides reached up to 96.2%, indicating that one of the mechanisms of disease suppression by *B. velezensis* FJAT-46737 was lipopeptide secretion.

The production of lipopeptide by *Bacillus* species is based on the strains: some strains can coproduce two or three classes of lipopeptides, while others can yield only one class [11–13]. *B. licheniformis* MB01 produces surfactin only [12], *B. subtilis* K1 coproduces surfactins and iturins, while *B. amyloliquefaciens* SYBC H47 yields three types of lipopeptides, namely, bacillomycin, fengycin and surfactin [36]. In the present study, the *B. velezensis* strain FJAT-46737 could coproduce three types of lipopeptides: C_14_–C_16_ iturin A, C_16_ fengycin A/B, C_16_ fengycin A_2_/ B_2_, as well as C_14_–C_15_ surfactin. Furthermore, the yield of lipopeptides was significantly affected by the medium components (such as carbon and nitrogen sources, trace metals, etc.) and cultivation conditions (such as culture temperature, incubation time, rotary speed, etc.) [37]. In this study, *B. velezensis* FJAT-46737 could yield three types of lipopeptides in all six common culture media (A ~ F). However, the antibacterial activities of the crude lipopeptides (10 mg/mL) produced from culture media A, B or F were much weaker than that of the other three media (C, D and E). The carbon source has been considered as the primary factor influencing the lipopeptide production. For example, Li et al. found that variations in carbon sources in the culture medium changed the type of lipopeptides produced by *B. licheniformis* HSN221 [38]. However, the types of lipopeptides produced by FJAT-46737 were not changed if the culture medium contained glucose or not. Moreover, nitrogen sources have been reported to play an important role in the regulation of biosurfactant synthesis [39]. Our results showed that rich organic nitrogen sources in the media were beneficial for producing fengycin and surfactin. Furthermore, yeast extracts had a greater influence on the production of fengycin than iturin and surfactin, which may be related to the less sensitive response of the enzymatic complex for fengycin biosynthesis to nitrogen sources compared with that for iturin and surfactin [40].

Interestingly, opposite effects were observed for the culture temperatures on the contents of the fengycins and surfactins generated by strain FJAT-46737: with the increasing temperature, the content of fengycins decreased while that of surfactin increased. These results were not consistent with the report by Monteiro et al., who found that low temperature (15 °C) was suitable for producing surfactins and the amount of fengycin was not affected by temperature changes [37]. Fengycin and surfactin are synthesized nonribosomally by fengycin synthetases and surfactin synthetases, respectively [41]. Usually, the optimum temperature for enzyme activity is 37 °C, which can explain why the content of surfactins increases along with increase of the culture temperature. The reduction of fengycin under high temperatures could attributed to the decreased activity of fengycin synthetases. Generally, the suitable temperatures for crop growth in the field are 20 °C–30 °C. Our results indicated that 25 °C was a favorable temperature for the production of lipopeptides producing the strongest antibacterial activities, implying that strain FAJT-46737 and its lipopeptides have good ecosystem adaptability and prospects for future agricultural application. Previous studies demonstrated that surfactins display antibacterial activities while iturins show strong antifungal activities with limited antibacterial activity. The fengycin family has been considered specific against filamentous fungi, although two cases recently reported by Villegas-Escobar et al. [14] and Chen et al., [42] indicated that fengycins exhibited strong antibacterial activity against *R. solanacearum* in vitro. In the present study, the correlation analysis between the cell-free supernatant antibacterial activities and lipopeptide contents indicated that the antibacterial activities of cell-free supernatant were significantly positive correlated with the content of fengycins. This finding is consistent with a report showing that a significant relationship occurred between the strong antibacterial activity and production of fengycin and surfactin of *Bacillus* isolates [43]. In addition, only the purified fraction SPE70 exhibited antibacterial activity in vitro, and it only consisted of fengycins, indicating that the antibacterial activity of the lipopeptide mixtures could be due to the fengycins. These results further confirmed the antibacterial activity of fengycins first reported by Villegas-Escobar et al. [14] and Chen et al., [42].

## Conclusions

A new *B. velezensis* strain FJAT-46737 with broad-spectrum antimicrobial activities was confirmed to have strong antibacterial activity against the bacterial wilt pathogen *R. solanacearum* by both in vivo and in vitro experiments. Moreover, suppressive effects of FJAT-46737 were associated with lipopeptide secretion, especially the content of fengycins. Therefore, FJAT-46737 and its lipopeptides have good application prospects for the biocontrol of bacterial wilt.

## Methods

### Strains and chemicals

Strain FJAT-46737 was isolated from a soil sample from the Huanggang Mountain area, Fujian Province, China, and preserved at the China General Microbiological Culture Collection Center (CGMCC No. 14661). The pathogenic strains *R. solanacearum* FJAT-91 (CGMCC No. 10692, tomato pathogen), *R. solanacearum* FJAT-77 (peanut pathogen), *Escherichia coli* FJAT-301, *F. oxysporum* f. sp. *capsicum* FJAT-831, *F. oxysporum* f. sp. *niveum* FJAT-30265 and *F. oxysporum* f. sp. *melonis* FJAT-9230 were preserved at the Agricultural Bioresources Research Institute, Fujian Academy of Agricultural Sciences, Fujian, China. All the culture media used for bacterial and fungal growth were purchased from AoBoXing Biological Technology Co., Ltd. (Beijing, China). The cultivation substrates used for the pot experiments were purchased from Xiamen Jiang Ping biological Technology Co., Ltd. (Xiamen, China). The reference standards for iturin and surfactin were produced from *B. subtilis* and purchased from Sigma (St. Louis, MO, USA).

### Antimicrobial spectra of the strain FJAT-46737

Using the agar disk diffusion method, strain FJAT-46737 was evaluated for its in vitro potential to inhibit several animal and plant pathogens, including *R. solanacearum*, *E. coli*, *F. oxysporum* f. sp. *capsicum*, *F. oxysporum* f. sp. *niveum* and *F. oxysporum* f. sp. *melonis*. For the fungal pathogen strains, a block of mycelium with a 7 mm-diameter was placed onto the center of a sterile potato dextrose agar (PDA) plate and then cultures of strain FJAT-46737 were streaked with sterilized toothpicks at a distance of 2.5 cm away from the margins of the mycelia colony and cultivated at 28 °C for three to 7 days. The PDA plate inoculated only with the pathogenic fungi was used as control.

The antagonistic effects of strain FJAT-46737 against the bacterial pathogenic strains were investigated using a two layer plating method. Semi-solid nutrition agar (NA in Table S1 for cultivating *R. solanacearum*)/Luria–Bertani (LB in Table S1 for cultivating *E. coli*) media containing pathogenic bacterial culture suspensions were poured onto Petri plates (90 mm) coated with the corresponding solid media. After solidification, a colony of the strain FJAT-46737 was streaked on the plate, and then cocultivated at 30 °C for 2 days. The NA/LB plate inoculated only with the strain FJAT-46737 was used as control. The inhibition rate was calculated according to the method descrbied by Chen et al. [44].

### Identification of strain FJAT-46737

Strain FJAT-46737 was streaked on NA agar plates and cultured at 30 °C for 48 h for investigating the colony morphology. The genomic deoxyribonucleic acid (DNA) of FJAT-46737 was extracted using the sodium dodecyl sulfate (SDS) method, detected by agarose gel electrophoresis and quantified by Qubit® 3.0. Strain FJAT-46737 was further identified based on an analysis of its 16*S rRNA* and partial gyrase subunit B (*gyrB*) gene sequences and whole-genome sequence. The 16*S rRNA* gene was amplified and sequenced using the universal primers 27F and 1492R. The *gyrB* gene was amplified using the specific primers F (5′-GAAGTCATCATGACC-3′) and R (5′-AGCAGGGTACGGAT-3′). Whole-genome sequencing was performed on an Illumina HiSeq PE150 platform at the Beijing Novogene Bioinformatics Technology Co., Ltd. The average nucleotide identity (ANI) was calculated using the OrthoANIu algorithm [45]. An ANI value cut-off of 96% is recommended for species delineation [30, 46]. The *16S rRNA* gene, *gyrB* gene and whole-genome sequences of strain FJAT-46737 were deposited in GenBank under the accession numbers MG924092, MH470338 and CP044133, respectively. The secondary metabolite gene clusters in strain FJAT-46737 were predicted using the antibiotics Secondary Metabolite Analysis Shell (antiSMASH) tool.

### Extraction and identification of the lipopeptides

FJAT-46737 was grown at 30 °C in 50 mL of culture medium (Table S1) in a shaking incubator (170 rpm). After 48 h, the culture was centrifuged at 6000 g for 5 min to remove the cells. The supernatant was adjusted to pH 2.0 with 3 mol/L HCl to obtain the crude lipopeptides. The extracted lipopeptides were dissolved in phosphate buffer and then lyophilized [42]. The obtained lipopeptide powder was dissolved in pure methanol for antimicrobial activity tests, for the qualitative and quantitative analyses and it was dissolved in water for the further pot experiments.

Qualitative and quantitative analyses of the lipopeptides were performed using liquid chromatography quadrupole time-of-flight tandem mass spectrometry (LC-QTOF-MS/MS) technology based on previous methods [42]. The structure of the lipopeptides was estimated by their accurate mass and MS/MS fragmentation patterns according to the literatures [47–51]. The content of each type lipopeptide was defined as follows: milligram of standard/liter of culture supernatant (mg/L).

### Biocontrol evaluation of FJAT-46737 against tomato bacterial wilt

Strain FJAT-46737 was cultured at 30 °C for 48 h in medium E (Table S1) in a shaking incubator (170 rpm). Tomato seedlings with 3–4 emerged leaves were transplanted into pots with a diameter of 15 cm at 30 °C. After 2 days, two groups (each with thirty seedlings) were drenched with 100 mL/pot of the whole FJAT-46737 cultures (10^8^ cfu/mL) and the double diluted culture supernatant. The lipopeptide-treated group consisted of thirty tomato seedlings that were carefully uprooted, the roots were then dipped in the crude lipopeptide solution (1 mg/mL, pH 6.5) for 1 h and then the seedlings were transplanted into pots. Each pot was drenched with 100 mL of the *R. solanacearum* cultures (10^8^ cfu/mL) after 3 days. Five control groups were also included: one was infected with only the *R. solanacearum* cultures and the others were irrigated with only water, whole cultures, supernatant and lipopeptides. Each treatment or control group was replicated three times. The survivability of the seedlings was monitored at a regular interval (2 days). The above pot experiments were carried out from June to August 2017 in a greenhouse with the following incubation conditions: light cycle of 16-h day-8-h night; temperature of 28–32 °C; and relative humidity of 60–80%. The strain FJAT-46737, supernatant, lipopeptide and water did not cause the happen of disease in tomato plants. Thus, the disease index (di), disease incidence (DI) and biocontrol efficacy of bacterial wilt were calculated according to the method described by Xiong et al. [39].

### Antimicrobial spectrum of the lipopeptides

The crude lipopeptide extracts from FJAT-46737 were dissolved in pure methanol to the final concentrations of 2.5–30 mg/mL. The antimicrobial activities of the crude lipopeptides were evaluated against eight pathogenic strains using the oxford cup method [52]. The pathogenic strains were grown at 30 °C in the corresponding liquid media for 48 h (for bacteria) /72 h (for fungi). A two layer plate containing fungal spores (10^5^ spores/mL) or bacterial culture suspensions (10^6^ cfu/mL) was prepared, and then four oxford cups were placed onto each plate and 150 μL of either the crude lipopeptides or cell-free supernatants was added. Methanol served as a negative control. Finally, the plates were cultivated at 30 °C for 48 h and the inhibition zone diameter was measured. All experiments were repeated three times.

### Effects of cultivation parameters on the antagonistic activities of the lipopeptides

*R. solanacearum* FJAT-91 was selected as an indicator strain. The culture media of A, B, C, D, E and F in Table S1 and temperatures of 20, 25, 30, 35 and 40 °C were selected for cultivating strain FJAT-46737. The antagonistic activities of the lipopeptides produced under different culture conditions were determined by the aforementioned agar disk diffusion method [42].

### Purification of antibacterial lipopeptides

The lipopeptides were loaded onto a solid phase extraction (SPE) C_18_ cartridge (6 g/60 mL) and washed with 60 mL of water and different proportions of methanol (10 to 100%). After evaporation, the obtained fraction was dissolved in water and used for the antibacterial activity test.

### Statistical analysis

An analysis of variance (ANOVA) of the quantitative data was carried out using the statistical software SPSS 19.0. Duncan’s multiple range tests were performed to determine the significant differences among the data (*p* < 0.05).

## Supplementary information

**Additional file 1: Figure S1.** The chemical structure of three types of cyclic lipopeptides. **Figure S2.** Morphology of *Bacillus* strain FJAT-46737. **Figure S3.** Phylogenetic tree based on the *16S rDNA* sequences showing the position of strain FJAT-46737 (accession number: MG924092). The type strains of *Bacillus* sp. and representatives of some other related taxa. Scale bar represents 0.001 substitutions per nucleotide position. It is note that the strain *B. amyloliquefaciens* subsp. *plantarum* FZB42 was renamed as *B. velezensis*. **Figure S4.** Phylogenetic tree based on the *gyrB* sequences showing the position of strain FJAT-46737 (accession number: MH470338). The type strains of *Bacillus* sp. and representatives of some other related taxa. Scale bar represents 0.001 substitutions per nucleotide position. **Figure S5.** The prediction of gene clusters of bioactive secondary metabolites in strain FJAT-46737. **Figure S6.** The antibacterial photo of lipopeptide (0.1 ~ 1 mg/mL) against *R. solanacearum* FJAT-91. **Figure S7.** The full scan LC–ESI–MS chromatogram of fraction SPE70. **Table S1.** Culture medium components

## Data Availability

All data generated or analyzed during this study are included in this published article and its supplementary information files. The *16S rRNA*, *gyrB* and whole genome sequences of this study have been submitted to NCBI GenBank database (accessions MG924092, MH470338 and CP044133).

## Abbreviations

CGMCC: China General Microbiological Culture Collection Center
PDA: Potato dextrose agar
LB: Luria–Bertani
NA: Nutrient agar
SDS: Sodium dodecyl sulfate
gyr B: Gyrase subunit B
DNA: Deoxyribonucleic acid
RNA: Ribonucleic acid
ANI: Average nucleotide identity
antiSMASH: Antibiotics Secondary Metabolite Analysis Shell
di: Disease index
DI: Disease incidence
SPE: Solid phase extraction
ANOVA: Analysis of variance
LC-QTOF-MS/MS: Liquid chromatography coupled to quadrupole time-of-flight tandem mass spectrometry
