# Supplementary material for: Biocontrol of tomato bacterial wilt by the new strain Bacillus velezensis FJAT-46737 and its lipopeptides
Source: BMC Microbiol. 2020 Jun 15;20:160. doi: 10.1186/s12866-020-01851-2 (PMC7296739; doi:10.1186/s12866-020-01851-2)
Supplement: Supplementary file 1 — Additional file 1: Figure S1. The chemical structure of three types of cyclic lipopeptides. Figure S2. Morphology of Bacillus strain FJAT-46737. Figure S3. Phylogenetic tree based on the 16S rDNA sequences showing the position of strain FJAT-46737 (accession number: MG924092). The type strains of Bacillus sp. and representatives of some other related taxa. Scale bar represents 0.001 substitutions per nucleotide position. It is note that the strain B. amyloliquefaciens subsp. plantarum FZB42 was renamed as B. velezensis. Figure S4. Phylogenetic tree based on the gyrB sequences showing the position of strain FJAT-46737 (accession number: MH470338). The type strains of Bacillus sp. and representatives of some other related taxa. Scale bar represents 0.001 substitutions per nucleotide position. Figure S5. The prediction of gene clusters of bioactive secondary metabolites in strain FJAT-46737. Figure S6. The antibacterial photo of lipopeptide (0.1 ~ 1 mg/mL) against R. solanacearum FJAT-91. Figure S7. The full scan LC–ESI–MS chromatogram of fraction SPE70. Table S1. Culture medium components [file 12866_2020_1851_MOESM1_ESM.docx]

**Biocontrol of tomato bacterial wilt by the new strain *Bacillus velezensis* FJAT-46737 and its lipopeptides**

**Meichun Chen, ^1^ Jieping Wang, ^1*^ Bo Liu, ^1^ Yujing Zhu, ^1^ Rongfeng Xiao^1^, Wenjing Yang, ^2^ Cibin Ge ^1^ and Zheng Chen ^1^**

^1^Agricultural Bioresources Research Institute, Fujian Academy of Agricultural Sciences, Fuzhou, 350003, China

^2^College of Biological Science and Engineering, Fuzhou University, Fuzhou, 350001, China

* Corresponding authors: Tel/Fax: +86059187864601. E-mail: [781063449@qq.com](mailto:781063449@qq.com) (Jieping Wang)


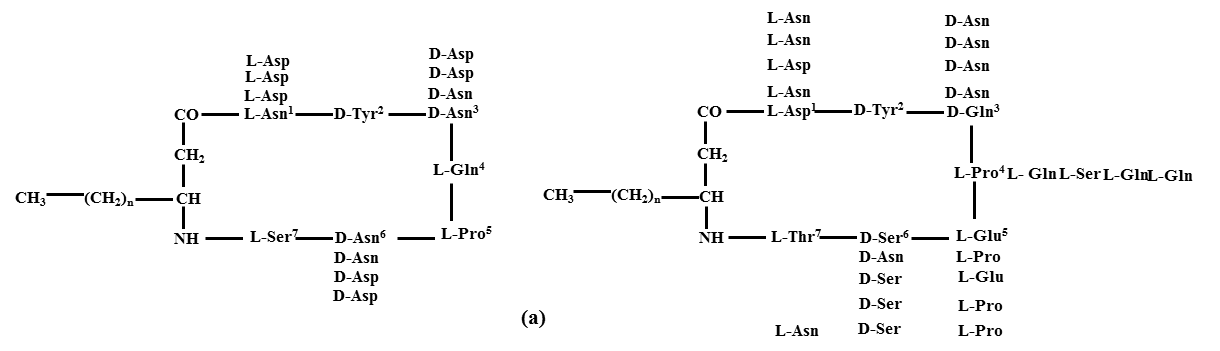


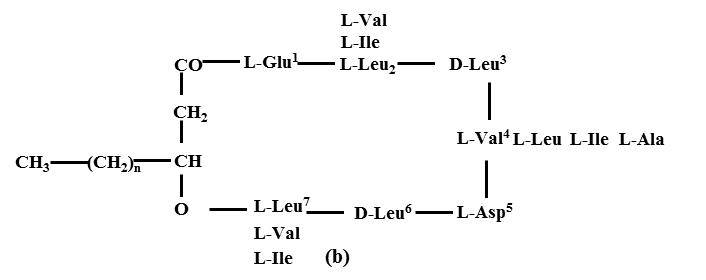


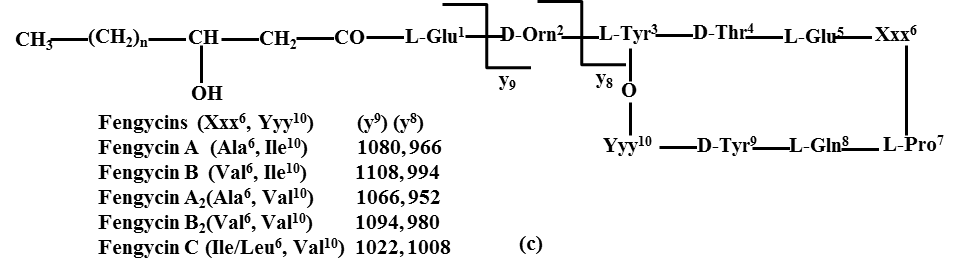


Figure S1. The chemical structure of three types of cyclic lipopeptides.


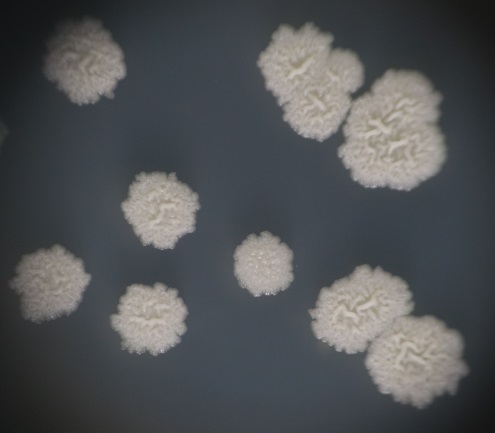


Figure S2. Morphology of *Bacillus* strain FJAT-46737


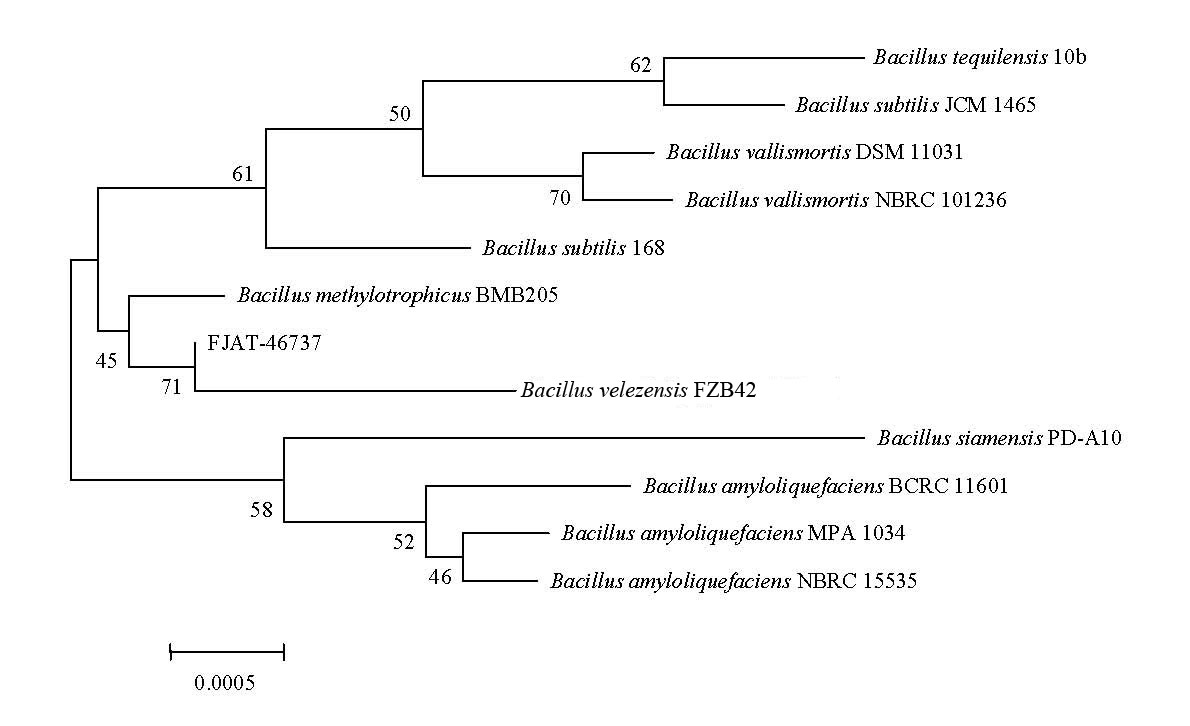


Figure S3. Phylogenetic tree based on the 16S rDNA sequences showing the position of strain FJAT-46737 (accession number: MG924092). The type strains of *Bacillus* sp. and representatives of some other related taxa. Scale bar represents 0.001 substitutions per nucleotide position. It is note that the strain *B. amyloliquefaciens* subsp. *plantarum* FZB42 was renamed as *B. velezensis*.


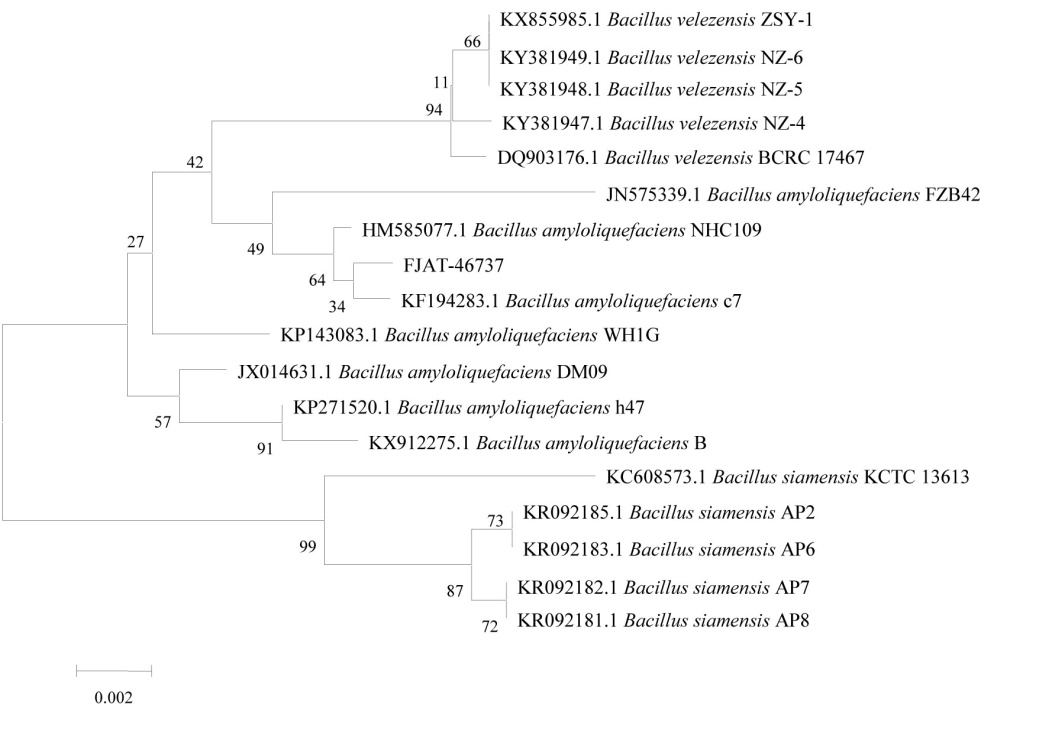


Figure S4. Phylogenetic tree based on the *gyrB* sequences showing the position of strain FJAT-46737 (accession number: MH470338). The type strains of *Bacillus* sp. and representatives of some other related taxa. Scale bar represents 0.001 substitutions per nucleotide position


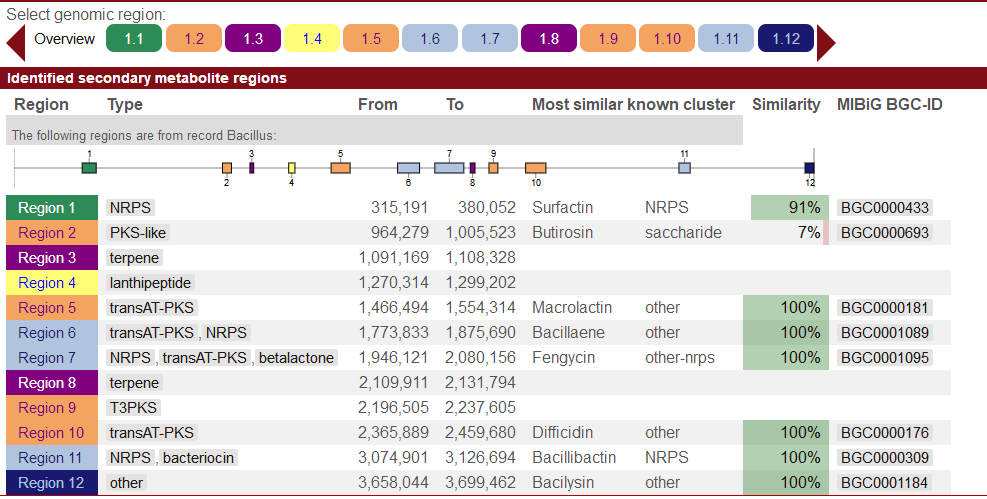


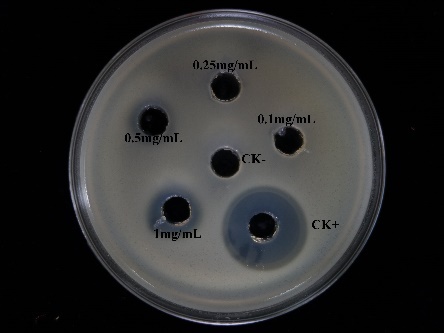
Figure S5. The prediction of gene clusters of bioactive secondary metabolites in strain FJAT-46737

Figure S6. The antibacterial photo of lipopeptide (0.1~1mg/mL) against *R. solanacearum* FJAT-91


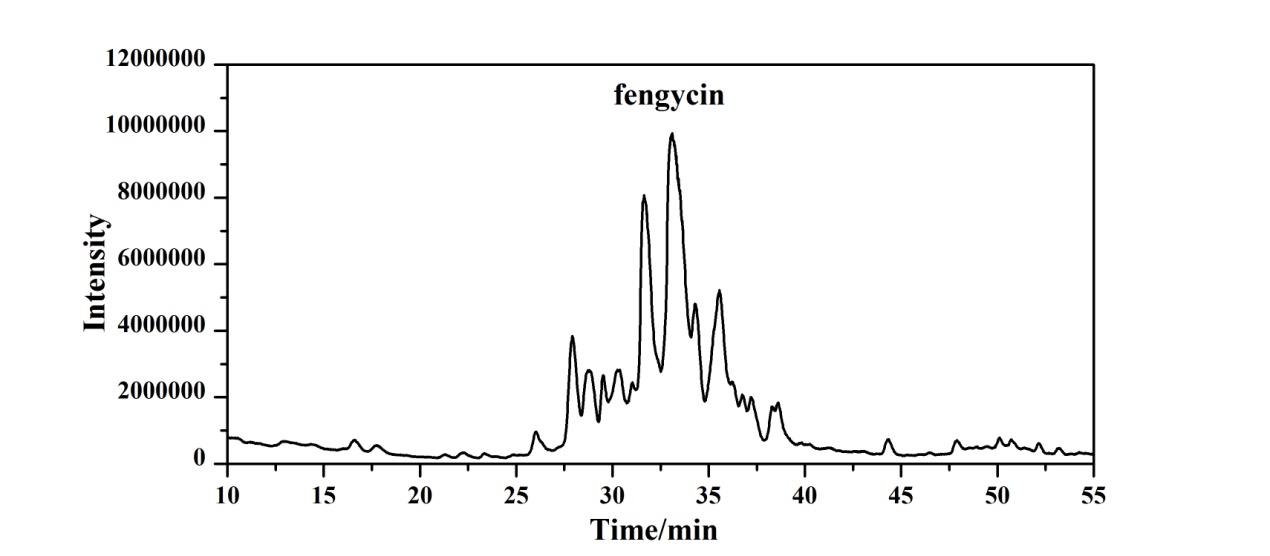


Figure S7. The full scan LC–ESI–MS chromatogram of fraction SPE70.

Table S1. Culture medium components

| No. | medium | culture medium components (g/L) |
| --- | --- | --- |
| 1 | A medium (LB) | tryptone 10, yeast extracts 5, NaCl 5 |
| 2 | B medium (NA) | beef Extract 3, peptone 5, glucose 10 |
| 3 | C medium | beef Extract 5, peptone 10, yeast extracts 5, NaCl 5, glucose 10 |
| 4 | D medium | beef Extract 5, peptone 10, yeast extracts 5, glucose 10 |
| 5 | E medium | Potato extract 5 peptone 10, NaCl 5, glucose 15 |
| 6 | F medium | beef Extract 8, yeast extracts 5, glucose 1 |
| 7 | PDA medium | potato, 200, glucose, 20, agar, 18 |
